# Determining the exact location of a public bicycle station—The optimal distance between the building entrance/exit and the station

**DOI:** 10.1371/journal.pone.0212478

**Published:** 2019-02-20

**Authors:** Shinan Shu, Yang Bian, Jian Rong, Dandan Xu

**Affiliations:** Beijing Engineering Research Center of Urban Transport Operation Guarantee, Beijing University of Technology, Beijing, China; University of British Columbia, CANADA

## Abstract

As a sustainable mode of transportation, public bicycles significantly improve daily mobility. The location of stations is a key element for the success of a public bicycle system, as a long walking distance will reduce people’s willingness to use this mode of transportation. Building forms in China are different from the open type seen abroad. Many residential, office and school areas are enclosed by walls, and pedestrian flow is concentrated at the entrances/exits of these areas. Therefore, the station must be located close to the building entrance/exit. Previous studies on station location located the stations only per zone, without providing the exact locations of the stations in the zones. This paper considers the optimal distance between the building entrance/exit and the station to determine the exact station locations. The results can serve as a reference for the planning and optimization of public bicycle stations. A questionnaire survey was conducted in Beijing to determine users’ walking distances to the stations. The results indicated that the walking distance decay laws of stations were different for different land uses. Moreover, a binary logistic model was developed to verify that users with different travel purposes have different walking distances. Based on the above results, we explored the optimal distances and tolerable distances between the building entrance/exit and the station for different land uses. These distances can be used to determine exact station locations to meet users’ physiological and psychological needs.

## Introduction

As the requirements of sustainable development, there has been increasing interest in improving the ecological environment and promoting the conservation of energy resources worldwide. Public bicycle systems (PBSs), which advocate green traffic, have been extensively researched in recent years [1]. Public bicycles can improve mobility, reduce congestion, lower emissions, and increase environmental awareness. Public bicycles can also be integral in bridging first-and-last-kilometer gaps in transportation networks and can encourage multi-modal trips [2].

PBSs have existed for nearly 50 years and have been increasingly used worldwide in the past ten years. China has become the country with the greatest number of public bicycles. PBSs have been built in 237 cities, and 750,000 public bicycles were in operation in 2015 [3]. The Beijing PBS was officially launched in June 2012. By the end of 2016, this PBS had built up a system consisting of over 67,000 bikes and 2,700 stations. Average daily use of the Beijing PBS has reached approximately 200,000, and the average daily usage rate is 4.1 times.

**Fig 1:**
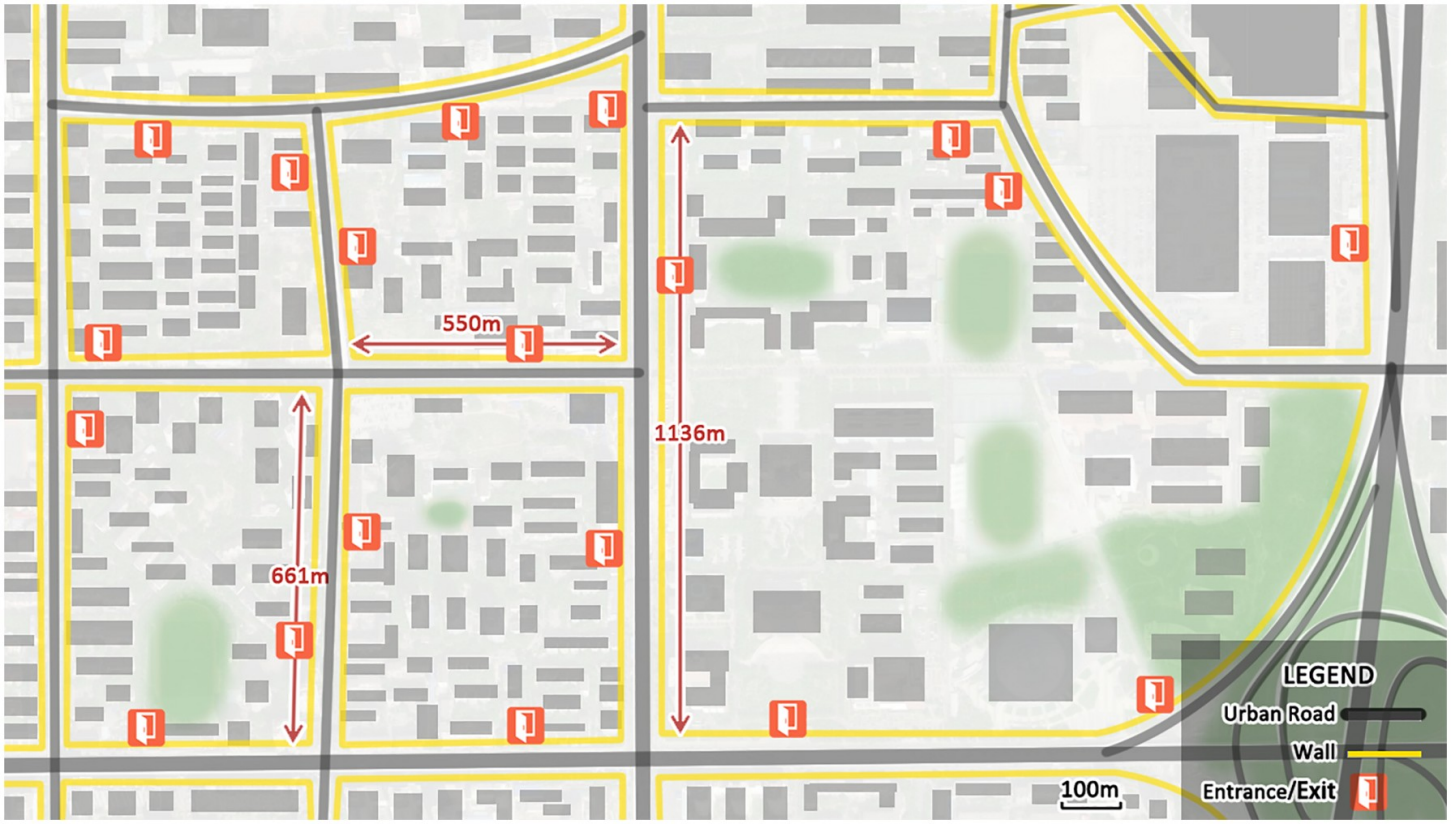
Building form in China: Closed network, long blocks and few building entrances/exits. (for illustrative purposes only).

**Fig 2:**
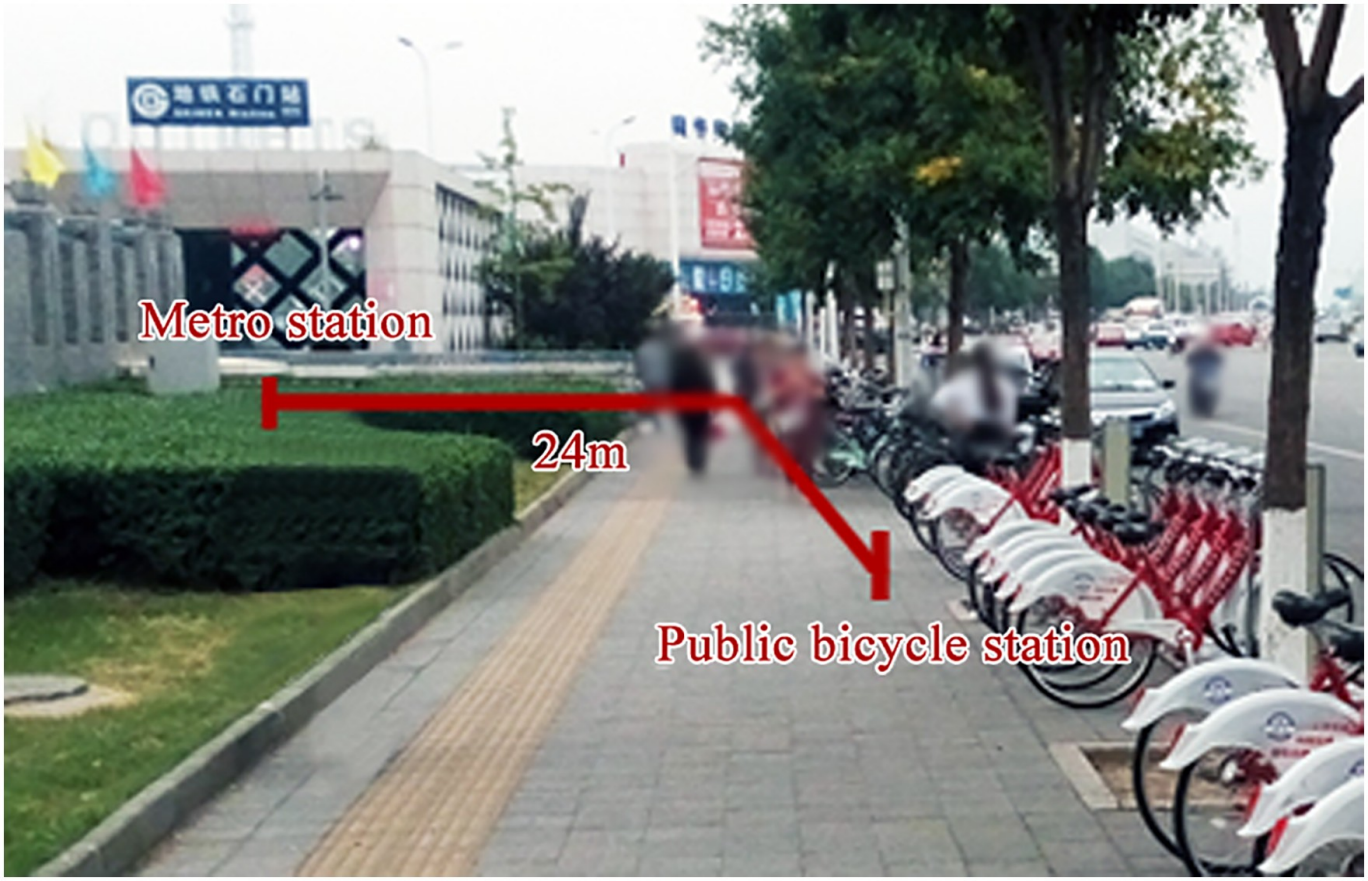
Walking distance between the public bicycle station and entrance/exit of the metro station.

Public bicycles have transformative effects on urban mobility and local planning. PBS planning is related to not only infrastructure construction but also land use management and urban design. Therefore, scientific PBS planning is important for improving livability and guiding future growth [2]. The Beijing PBS planning scheme presents the overall construction scale, station location and locking device scale of each station. First, the overall construction scale is determined according to the budget. Second, the approximate location and scale of each station will be confirmed, with metro stations and residential, commercial and office areas being the typical key areas. Finally, the exact locations of the stations are determined. Following the principle of “close setting”, stations will be set on a sidewalk close to the building entrance/exit. The building form in China is different from the open type seen abroad. As shown in Fig 1, many buildings and places, such as residential areas, office areas and schools, are enclosed with walls. Thus, there are only a few entrances/exits to these areas, causing pedestrian flow to be concentrated at the entrance/exit. Commercial buildings have few entrances/exits and high pedestrian flow, particularly at metro stations. People must walk long distances to the entrances/exits. Therefore, public bicycle stations must be set close to the building entrance/exit (including residential areas, office areas, schools, commercial buildings and transportation buildings). As shown in Fig 2, the public bicycle station is located 24 m away from the metro station entrance/exit. However, there is typically a longer distance from the building entrance/exit due to space and electricity restrictions. A long walking distance decreases users’ willingness to use a public bicycle; they will refrain from using a public bicycle if it is over a certain distance away. Hence, the distance between the building entrance/exit and station should have an upper limit to guarantee that users can reach services within acceptable walking distances.

This paper explores the optimal distance between building entrances/exits and PBS stations to determine the exact location of these stations. First, a questionnaire survey of different stations considering land use, environment and usage rates was conducted to obtain the demographics, travel characteristics, and walking distances of individuals. Second, the walking distance decay law of users’ station selection was further investigated from two aspects: the distance decay laws of different types of land uses and the distance decay laws of different land use intensities. Third, a binary logistic model was constructed to understand how differences in socio-demographic characteristics, travel characteristics and psychological feelings between individuals explain their different walking distances. Finally, the optimal distances and tolerable distances of different types and intensities of land uses were obtained by establishing a nonlinear regression model. The conclusion of this paper provides effective guidance for determining the exact station location and is useful for applications.

## Literature review

The majority of previous and current studies related to public bicycles have focused on the history and benefits of PBSs [1,4–5], cycling-related safety issues [6–8], the rebalancing of bikes among public bicycle stations [9–10], and investigating cyclists’ preferences [11–12]. However, relatively few studies have considered the station location problem for PBSs. One of the reasons that residents are willing to use public bicycles is their convenience [13–14], and the unreasonable location of stations has a negative impact on convenience. Therefore, it is important to study the problem of locating public bicycle stations.

Facility location models have been widely applied to solve the problem of site selection. Their decision variables include the location, capacity and coverage, minimization of the intended distance between facilities and users, transportation cost minimization or coverage maximization [15–16]. Several scholars have applied the model to the study of the problem of public bicycle station allocation. Lin and Yang [17] considered the strategic planning of a PBS with service-level considerations. Considering the interests of both users and investors, an optimization model including a nonlinear integral and greedy heuristic was established to determine the number and locations of public bicycle stations, the network structure of bike paths connecting the stations, and the travel paths for users between each origin-destination pair. Martinez et al. [18] presented a mixed-integer linear program performed through a heuristic to optimize the location of stations assuming a fleet dimension and bicycle relocation calculation for a regular operating day. The main goal of the method is to maximize revenue. Chen and Sun [19] proposed a mathematical model to formulate the layout of public bicycle stations with the objective of minimizing investment budget constraints and users’ total travel time. This model can be used to determine the number and locations of the stations and the number of bikes and parking lockers at each station.

Other methodologies have also been used to define the locations of stations. García-Palomares et al. [20] proposed a Geographic Information System (GIS)-based method to analyze the spatial distribution of the potential demand for bike trips and applied location-allocation models to locate public bicycle stations. Dell et al. [21] established a model for the layout of stations based on GIS to serve the highest possible population. He found that the stations with higher user frequencies were always near transportation hubs, residential areas or commercial areas. Romero et al. [22] proposed a simulation–optimization method that related public bicycles to private cars to optimize the location of stations and performed an example verification in Santander, Spain. From the perspective of residents’ travel demand and traffic facility supply, He et al. [23] established a bi-level programming model to combine a self-adaptive genetic algorithm, the combined feedback modal-split and the assignment model. The optimal layout plan of stations can minimize the regional travel cost and the construction cost of PBSs. Lin et al. [24] used a graph convolutional neural network approach to predict station-level hourly demands in a large-scale bike-sharing network. Li and Wang [25] applied complex network theory to convert the station network into a topology structure, determined the important station hubs, and proposed a method of optimizing a complex network of urban public bicycle stations.

All these studies provide the background for our study, but they all miss an aspect of the real implementation of PBSs: the proximity of a public bicycle station to destinations. Shaheen et al. [26] conducted a two-year multi-national North American study of public bicycles. They interviewed operators, experts, and other stakeholders about station locations and station spacing. After evaluating the station locations, eleven operators indicated that the appropriate distance between a public bicycle station and public transit was an average of 120 meters, while other five programs stated that the public bicycle station needed to be as close as possible to public transit. In addition, fifteen programs indicated the optimal distance between stations was 200 meters.

Most previous studies focused on the problem of locating public bicycle stations from a macro perspective; thus, they sought to determine the overall distribution by analyzing the entire scale, service radius and service object. Only a few studies have presented the optimal distance between public bicycle stations and public transit according to the practical experience of operators. Most of previous studies determined only the station’s approximate location; their methods specify the number of stations per zone without giving the exact locations of the stations inside these zones. The optimal distance between the station and building entrance/exit cannot be determined according to the user’s behavior. However, the approaching degree between the station and user is a crucial factor for the willingness to use a public bicycle [27–30]. There is a clear threshold for a user’s walking distance, and once beyond the tolerable distance, the user will choose to not use a bicycle. Therefore, the optimal distance between the station and building entrance/exit in actual project construction must be further understood. In addition, considering space and electricity restrictions, there should be an adjustable range of acceptable distances. To solve the above problems, this paper studied the travel characteristics and behavior rules of public bicycle users to obtain the optimal distance and tolerable distance for station location. Based on those results, the exact location for public bicycle stations was determined. The results of this paper can enable public bicycle station planning to better conform to users’ physiological and psychological needs.

## Data and method

### Land use classification

People with different travel purposes always have different travel demands. Users’ choice behaviors regarding public bicycle stations may also differ for different land uses. Therefore, this study classified land uses to explore the station location problem for different land uses. Because the attractive radius of a station is small (190 m) [31], a station is considered to be placed in one land use. The buildings served by station should be within the attractive radius; thus, the buildings are considered to be located in the same land use as the station.

**Fig 3:**
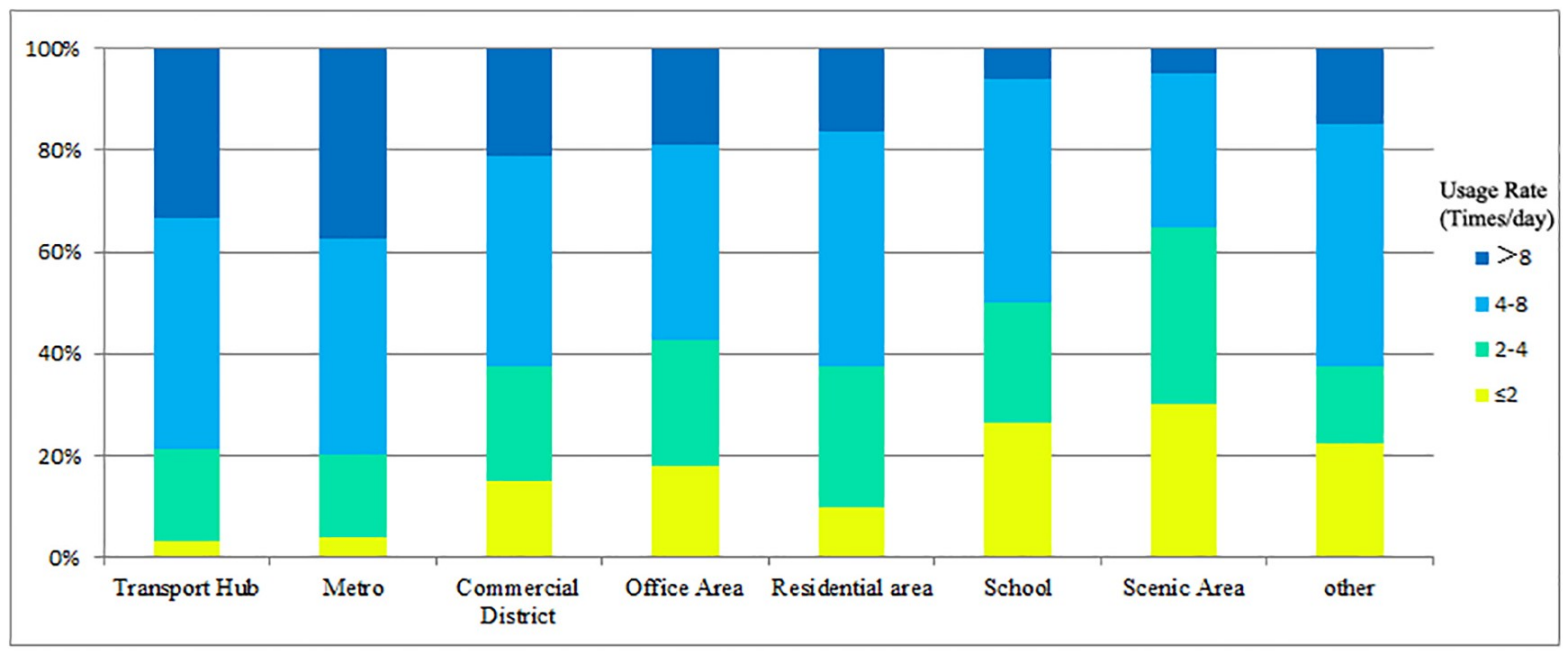
Usage rates of different types of stations. (Data from Beijing public bicycle information system).

Public bicycle stations are located in 8 types of land uses in Beijing, namely, transportation hub, metro, residential area, office area, commercial district, scenic area, school and other. The usage rates of the different types of stations are shown in Fig 3. Stations have a higher usage rate around transport hubs and metros and a lower usage rate around scenic areas and schools. The operation effect of stations differs significantly in different land uses; thus, the station location problem must be investigated for different land uses.

Shu et al. [32] found that in addition to the type of land use, the intensity of land use also has an effect on the operation effect of stations. The usage rates of stations around a given type of land use differ significantly; one of the key factors for the usage rate is the intensity of the land use. To determine the optimal distance between a station and building entrance/exit in different land uses, this paper classified land uses according to their type and intensity, with reference to Code for Classification of Urban Land Use and Planning Standards of Development Land (GB50137-2011) [33]. As shown in Table 1, based on the actual situation of Beijing, we divided the land uses into 7 categories according to type and into 16 categories according to intensity. According to the actual situation in China, colleges are more intense than middle/primary schools, and office buildings are more intense than research institutions. Therefore, within each land use type, the land uses in the first row are more intense.

**Table 1:** Land use classification.

Type	Intensity
Transport hub & metro	Combined transport hub
	Metro station
	Bus hub
Residential area	Large scale
	Mid-scale
	Small scale
Commercial district	Shopping mall
	Supermarket
Office area	Office building
	Research institutions
School	College
	Middle & primary school
Scenic area & park	Scenic spot
	Park
Other	Large amenity
	Small amenity

### Ethics statement

A questionnaire survey was conducted in Beijing. The cover of the questionnaire gave a detailed explanation of the objective of the survey. If users did not agree to take part in this survey, they could freely choose not to respond any questions without any consequences. Thus, we considered the receipt of a questionnaire to indicate informed consent. We didn’t obtain any data of personal identifiers and analyzed the data anonymously. The study was reviewed and approved by the Ethics Committee of College of Life Science and Bioengineering, Beijing University of Technology.

### Survey data

**Fig 4:**
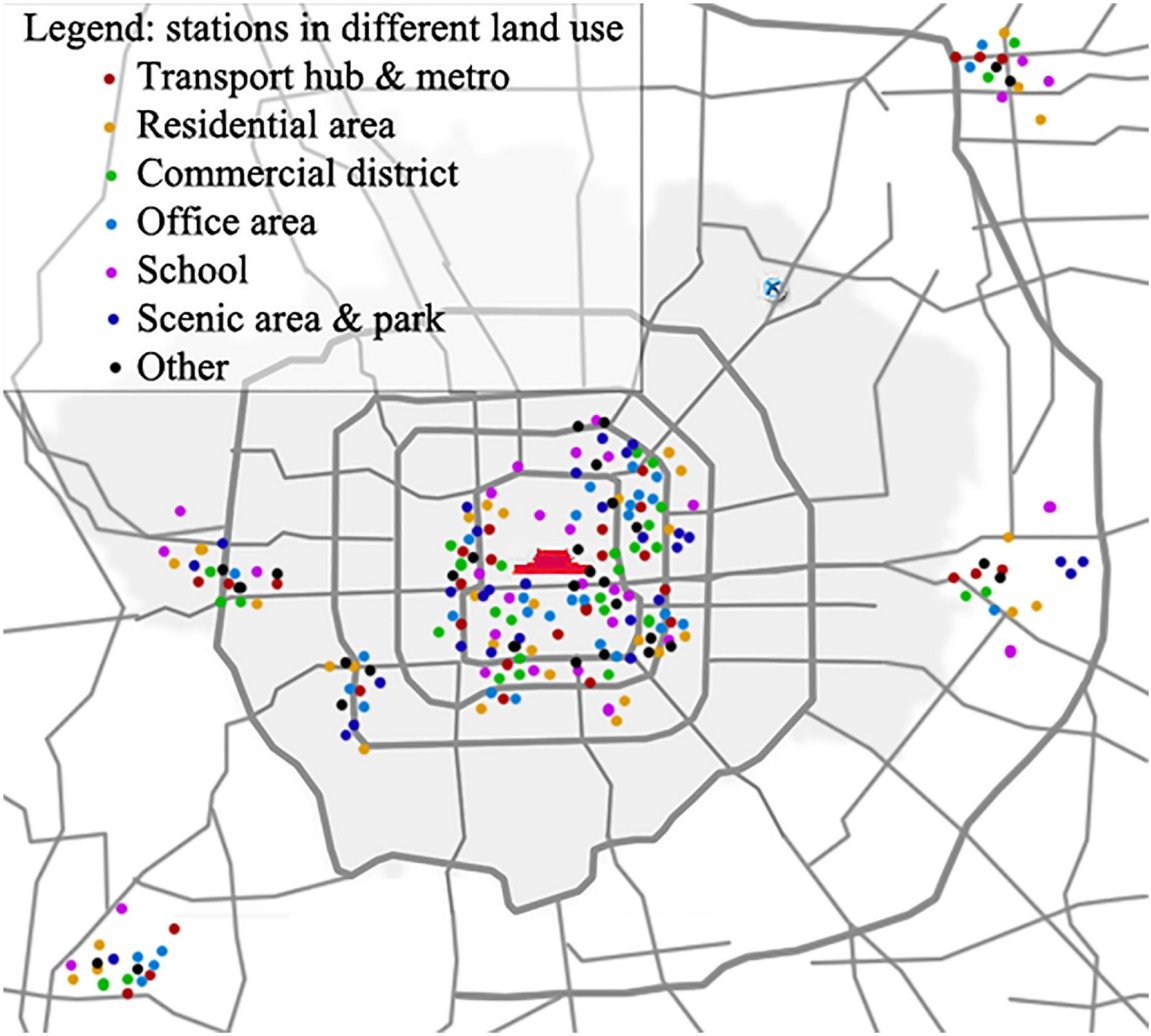
Locations of the survey stations. Station in different land use. (for illustrative purposes only).

The questionnaire survey was conducted in 220 public bicycle stations in Beijing, accounting for 12% of the total stations in 2015. Using the stratified sampling method, the survey stations were selected from eight districts of Beijing, including the Dongcheng district, Xicheng district, Chaoyang district, Fengtai district, Shijingshan district, Shunyi district, Tongzhou district and Fangshan district. These stations include 16 types of land uses and exhibit usage rates ranging from 0.2 to 18.6 times per day. The locations of these stations are shown in Fig 4.

The questionnaire survey was conducted from September 21 to September 27, 2015. A random distribution of 3,300 questionnaires yielded a return of 2,831 valid questionnaires. The number of valid questionnaires by land use was list in Table 2.

**Table 2:** Number of valid questionnaires by land use.

Type	Intensity	Number of valid questionnaires	Proportion
Transport hub & metro	Combined transport hub	162	6%
	Metro station	173	6%
	Bus hub	157	6%
Residential area	Large scale	162	6%
	Mid-scale	159	6%
	Small scale	150	5%
Commercial district	Shopping mall	180	6%
	Supermarket	176	6%
Office area	Office building	178	6%
	Research institutions	201	7%
School	College	180	6%
	Middle & primary school	203	7%
Scenic area & park	Scenic spot	188	7%
	Park	183	6%
Other	Large amenity	191	7%
	Small amenity	188	7%

The variables were generated from the 11 question items on the survey. As shown in Table 3, the variables were grouped into three categories. The items selected were based on previous research, and they are related to factors affecting the perception of public bicycle uses:

**Table 3:** Explanatory variables and descriptive statistics.

Variable	Description	No. obsv.	Mean
a1 Age	1 if under 24; 2 if 25–34; 3 if 35–44; 4 if 45–54; 5 if over 54.	329	2.856
a2 Monthly income	1 if below 3,000 yuan; 2 if 3,000–8,000 yuan; 3 if over 8,000 yuan.	459	2.046
a3 Car ownership	1 if own a car; 0 if without a car.	1,387	0.490
b1 Work/study	1 if uses public bicycle to commute to place of work or study; 0 otherwise.	1,554	0.549
b2 Shopping	1 if uses public bicycle for shopping; 0 otherwise.	1,067	0.377
b3 Leisure/sport	1 if uses public bicycle for leisure or sport; 0 otherwise.	210	0.074
b4 Usage frequency	1 if use public bicycle frequently; 0 otherwise.	2,301	0.813
b5 Cycling mode	1 if combines public bicycle with some other type of public transport; 0 if uses public bicycle alone for journeys.	1,627	0.575
b6 Cycling time	1 if in 20 minutes; 0 if more than 20 minutes.	2,112	0.746
b7 Walking distance	The actual walking distance (m) between the origin/ destination and the station asked.	2,831	124
c1 Degree of satisfaction	1 if satisfied; 2 if generally; 3 if dissatisfied.	1,438	1.542

Individual demographics: We asked users to provide their individual characteristics, such as age (variable a1) [31, 34], economic level (variable a2) [34, 35] and car ownership (variable a3) [36, 37].

Travel characteristics: following prior research, such as Tang et al. [34,38], work and school commuting (variable b1), shopping (variable b2), leisure and sport (variable b3) were considered, and following Fishman et al. [39–41], among others, other use-related variables were included, such as usage frequency (variable b4), cycling mode (variable b5), cycling time (variable b6) and the walking distance between the building entrance/exit and station (variable b7).

Psychological feeling: In previous research, Castillo-Manzano et al. [42] considered users’ degree of satisfaction with PBSs (variable c1). This variable could help to understand users’ perceptions of the PBS.

## Results and discussion

### Descriptive results

The number of observers and the means of the various factors are provided in Table 3. The value of the "No. obsv" for variable “walking distance” is the number of all observers. The values of the "No. obsv" for other variables are the number of observers when the variables are 1. In addition, the proportions of different options of questions were also counted. As demonstrated by the individual characteristics, the proportion of users from age 25 to 44 is as high as 60%; the proportion of users earning from 3,000 to 8,000 yuan monthly is approximately 63%; and 49% of users own a car. This indicates that public bicycles are mainly used by young and middle-aged individuals as well as middle-income individuals. In addition, residents who own a car do not reject the use of public bicycles.

The travel characteristics were tallied, including travel purpose, cycling mode, cycling time and actual walking distance. In terms of travel purpose, work/study trips account for 55%, living trips, such as shopping and dining out, account for 38%, and leisure/sport trips account for 7%. In terms of use frequency, 81% of responders use a public bicycle as a daily travel mode, 41% of them use it nearly every day, and 40% of them use it several times a week. In terms of cycling mode, 58% of responders combine a public bicycle with another type of public transport demonstrating that public bicycles play a significant role in solving the "last kilometer" problem. In terms of cycling time, 75% of responders complete their travel within 20 minutes, and the average cycling time was 17.5 minutes. This means that a public bicycle is mainly used for short-distance travel. In terms of walking distance, 70% of responders’ walking distances are within 100 m, and 40% of responders’ walking distances are between 100 m and 200 m. However, only 5% of their walking distances are more than 200 m. The average walking distance is 124 m.

In terms of psychological feeling, users had a relatively high satisfaction with PBSs. A total of 52% of responders indicated that they were satisfied, whereas 26% indicated that they were dissatisfied. A total of 86% of responders considered that the distance between the building entrance/exit and the station had a considerable influence on their satisfaction.

### Walking distance decay law

**Fig 5:**
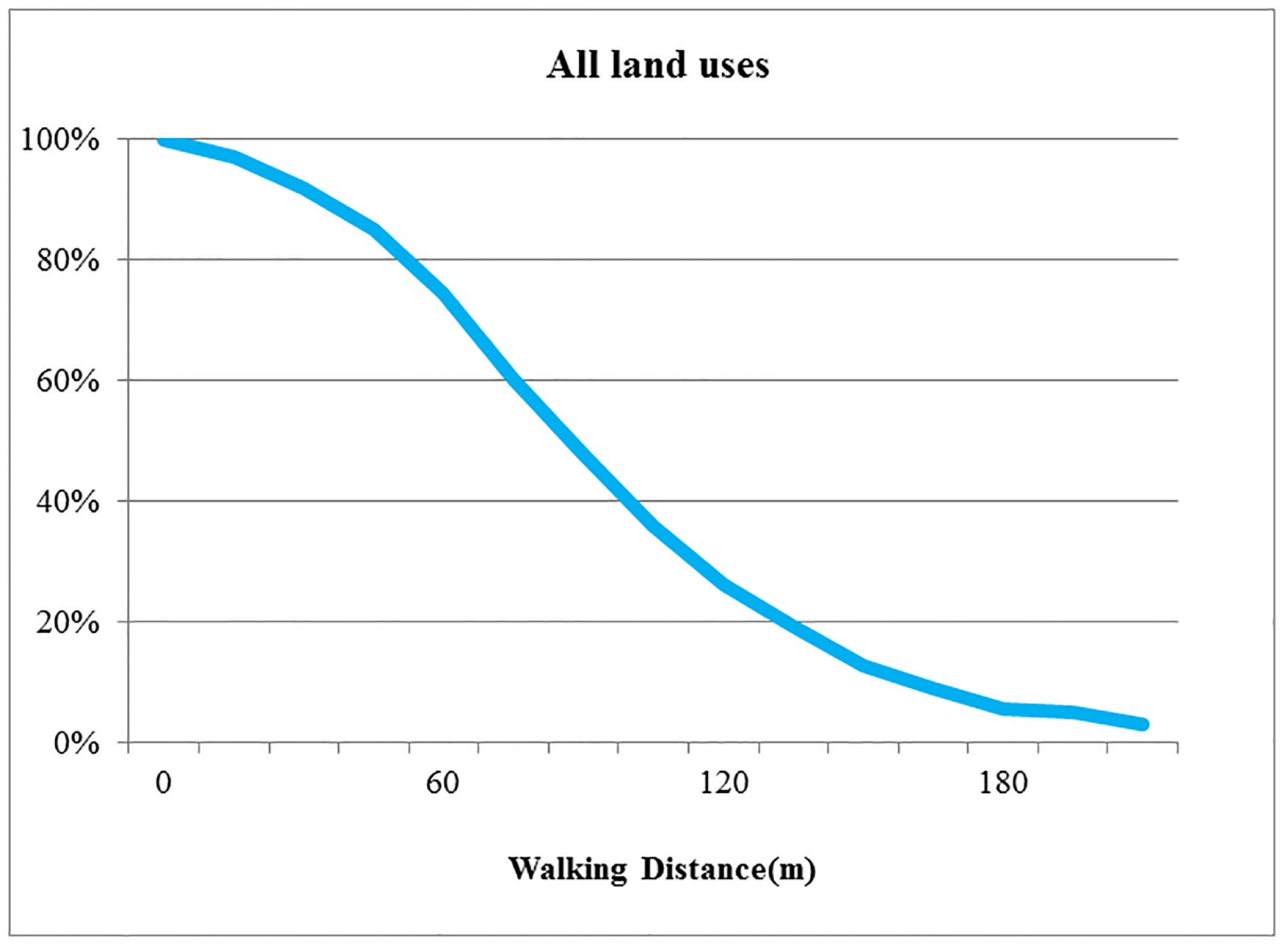
Walking distance decay graphs for all land uses. Percentage of users within a given walking distance.

To explore the behavior rules of station selection, walking distance decay graphs were drawn based on the survey data to analyze the relation between walking distance and users’ station selection probability. Fig 5 shows the general situation of user’s choice of station for all types of land uses. The curve represents the percentage of users who are willing to reach the station within a given walking distance, which is the percentage of users whose walking distance to the station is not more than the given distance. The analysis of the walking distance decay graph of all land uses demonstrates the following. First, when the walking distance is more than 200 m, the percentage of users is only 5%. This result means that few users are willing to use the stations that are more than 200 m away from the origin/destination. Second, the curve has a descending trend, which means that the number of users decreases with increasing walking distance from the building entrance/exit to the station. Third, as the walking distance increases, the decay of the number of users is initially rapid and then becomes more gradual. After 60 m, the curve declines rapidly with increasing distance. After 150 m, it declines more gradually with increasing distance. The number of users decreases rapidly when the distance is between 60 m and 150 m. It means the station has better set within 60 m away from the building entrance/exit.

Previous studies have determined the walking distance decay law of amenity selection. Resident’s willingness to use an amenity decreases with increasing walking distance [31]. The statistical results regarding walking distances shown above demonstrate that a user’s choice to use a public bicycle station follows the same law. This paper further studied the walking distance decay law of users’ choice of station from two aspects, including the laws of different types of land uses and the laws of different intensities of land uses.

#### Different types of land uses

**Fig 6:**
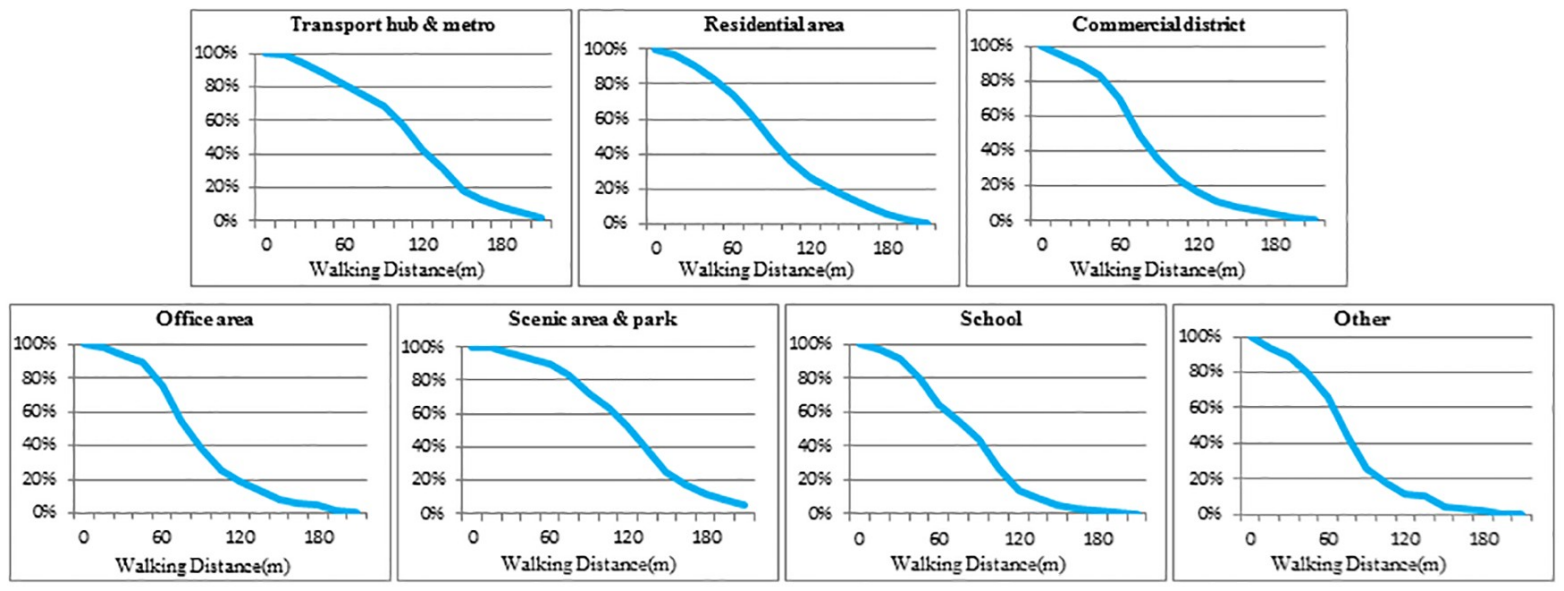
Walking distance decay graphs of different types of land uses. Percentage of users within a given walking distance.

The walking distance decay graphs of 7 categories of land uses are drawn in Fig 6 to explore the walking distance decay laws of different types of land uses. The walking distance decay graphs demonstrate that different types of land uses have different distance decay laws. The rapid decay interval of walking distance is different for different land uses, as is the percentage of users in the same walking distance interval.

The decay speed is slowest within a short distance of a scenic area or park. The number of users does not decay within 30 m, and it does not decay rapidly until reaching 100 m. Moreover, there are still many users that are willing to choose the station with a longer walking distance. When the walking distance is 150 m, the percentage is 15% for scenic areas and parks. The decay speed within a short distance of a transport hub and metro is also slow. The number of users decays rapidly when the distance reaches 60 m. When the walking distance is 150 m, 18% of users choose the station when located in a transport hub or metro.

The decay speed is the fastest within a short distance of schools and other land uses. The number of users decays rapidly when the distance is over 30 m, and the decay speed does not slow until the walking distance is 120 m. Only 6% of users are willing to choose the station when the walking distance is 150 m. For other land uses, the fastest decay interval is 30 m to 100 m. When the walking distance is 150 m, only 4% of users choose the station. Hospitals are the majority of land uses classified as “other” that have PBSs in Beijing. The main reason for the faster decay speed for other land uses is that people who go to the hospital have a higher focus on convenience, which leads to a shorter walking distance; thus, the stations located close to a hospital require a shorter walking distance.

#### Different intensities of land uses

**Fig 7:**
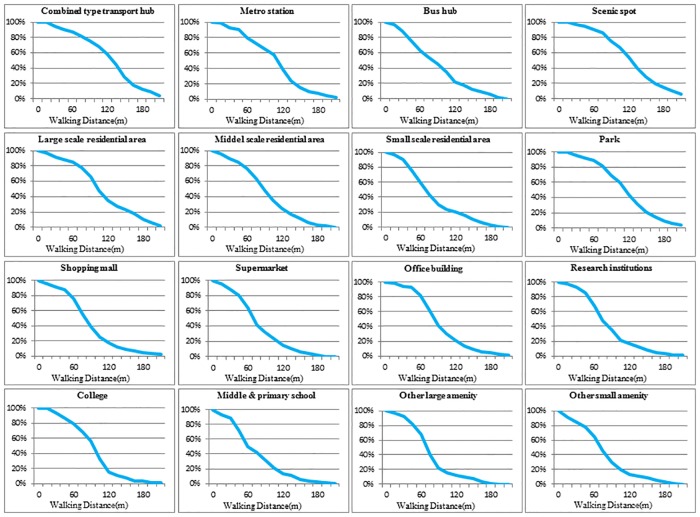
Walking distance decay graphs of different intensities of land uses. Percentage of users within a given walking distance.

The walking distance decay graphs of attraction points of different intensities are displayed in Fig 7. Taking residential areas as an example, large-scale residential areas have the slowest decay speed within a short distance. The number of users decays rapidly when the distance is greater than 90 m. For mid-scale residential areas, the number of users decays rapidly after 60 m. Small-scale residential areas have the fastest decay speed. The rapid decay starts when the distance is over 40 m. Moreover, the percentage of users is different for different intensities of residential areas when the distance is 150 m. The percentage is 23% for large-scale residential areas, whereas it is 12% for mid-scale residential areas and 10% for small-scale residential areas.

The walking distance decay graphs of the 7 types of land uses illustrate that land uses of other types exhibited the same rule under different intensities. These results indicate that land uses of different intensities have different distance decay laws. The decay speeds of high-intensity land uses are slower, and a longer walking distance will be accepted by users for higher intensity land uses.

### Walking distance affected by individual characteristic and travel characteristic

Different groups of people and people with different travel purposes will accept different walking distances. A binary logistic model was established to analyze the influencing degree of every factor. The walking distance was divided into two categories: less than 100 m and more than 100 m [31]. The binary variable of the model is whether a user’s walking distance exceeds 100 m. The influences of individual demographics, travel characteristics and degree of satisfaction on walking distance were analyzed. Table 4 presents the logistic estimation for the factors that determine whether a user’s walking distance is more than 100 m. Because estimated coefficients in logistic models cannot be interpreted directly, the marginal effects have been calculated at the mean.

**Table 4:** Logistic estimation of the marginal effects at the mean of PBS users’ walking distance after 100 m.

Variable	Walking distance Logistic regression	
	Odds ratios	Marginal effects
a1 Age	0.997	▽0.071%
a2 Monthly income	1.003	Δ0.071%
a3 Car ownership	0.633***	▽11.204%***
b1 Work/study	1.529**	▽10.404%**
b2 Shopping	1.667***	▽12.554%***
b3 Leisure/sport	1.812***	Δ15.835%***
b4 Usage frequency	1.471***	Δ8.625%***
b5 Cycling mode	0.852*	▽3.845%*
b6 Cycling time	1.420***	▽8.558%***
c1 Degree of satisfaction	1.177**	Δ3.771%**
No. observations		2,831
Log. Pseudo-likelihood		-860.609
Pseudo R2		0.380
Wald Chi2		77.011

Notes: 0.05<*p≤0.1.

0.01<**p≤0.05.

***p≤0.01.

Table 4 shows the explanations for the determinants that motivate a public bicycle user to accept a longer walking distance. In terms of individual characteristics, the decision to walk longer does not appear to be influenced by age (a1) or income (a2). The users who do not own cars (a3) are willing to walk longer to reach the stations, which suggests that the station should be located within a short distance from the building entrance/exit to attract more car travelers to transfer to a public bicycle.

In terms of travel purpose, users who are traveling for work/study (b1) and shopping (b2) accept a shorter walking distance, likely because both commuter and shopping travelers are more concerned with convenience and travel time. Users who are traveling for leisure/sport (b3) accept a longer walking distance. Thus, the distance between the station and entrance/exit of office and commercial areas should be shorter, whereas the distance between the station and entrance/exit of scenic areas and parks can be slightly longer. These results support the analysis results for the walking distance decay laws of different types of land uses.

In other respects, users who use public bicycles frequently (b4) and have a long cycling time (b6) accept a longer walking distance, indicating that residents who habitually use public bicycles are willing to choose stations even if they were located slightly farther away. The correlation between cycling model (b5) and walking distance is not as significant as other factors. Furthermore, the degree of satisfaction (c1) with PBSs is significantly correlated with walking distance; a short walking distance will help to improve users’ satisfaction.

### Optimal distances and tolerable distances for determining the exact station location

In actual project construction, the distance between the station and building entrance/exit should be understood to determine the exact location of stations. If the station location has does not have space or electricity constraints, the distance should be set as the optimal distance. If the location of the station is limited by space or electricity, the distance can be longer than the optimal distance. However, the distance between the station and building entrance/exit cannot be longer than the tolerable distance; otherwise, people will not choose the station. As noted above, the walking distances required to reach a station are different in different land uses, and the walking distances of people with different travel purposes are different. To meet the requirements of practical engineering, the optimal distances and tolerable distances of 16 types of land uses were obtained, and nonlinear regression models were developed (Table 5).

**Table 5:** Optimal distance and tolerable distance between the station and building entrance/exit.

Type	Intensity	Model	R^2^	Optimal distance (m)	Tolerable distance (m)
Transport hub & metro	Combined transport hub	y = 2.0220 ∙ 10^−7^x^3^ − 7.5660 ∙ 10^−5^x^2^ + 0.0020x + 0.9730	0.982	65	165
	Metro station	y = 2.9230 ∙ 10^−7^x^3^ − 9.0840 ∙ 10^−5^x^2^ + 0.0020x + 0.9830	0.981	55	165
	Bus hub	y = 1.2190 ∙ 10^−7^x^3^ − 2.1800 ∙ 10^−5^x^2^ − 0.0060x + 1.0331	0.986	30	130
Residential area	Large scale	y = 2.9630 ∙ 10^−5^x^3^ − 0.0022x^2^ + 0.017x + 0.9132	0.992	60	165
	Mid-scale	y = 2.6780 ∙ 10^−7^x^3^ − 7.1991 ∙ 10^−5^x^2^ + 0.0010x + 1.0041	0.994	45	150
	Small scale	y = 1.1621 ∙ 10^−5^x^3^ − 0.0001x^2^ − 0.0402x + 1.1486	0.976	35	140
Commercial district	Shopping mall	y = 3.2128 ∙ 10^−5^x^3^ − 0.0021x^2^ − 0.0028x + 1.0408	0.982	50	125
	Supermarket	y = 2.2192 ∙ 10^−5^x^3^ − 0.0010x^2^ − 0.028x + 1.1013	0.977	40	120
Office area	Office building	y = 3.3430 ∙ 10^−7^x^3^ − 8.8090 ∙ 10^−5^x^2^ + 0.0010x + 1.0331	0.982	45	140
	Research institutions	y = 2.5926 ∙ 10^−5^x^3^ − 0.0013x^2^ − 0.0211x + 1.1208	0.982	45	125
School	College	y = 4.4431 ∙ 10^−5^x^3^ −0.0032x^2^ + 0.0199x + 0.9147	0.987	50	120
	Middle & primary school	y = 1.1211 ∙ 10^−5^x^3^ − 0.0002x^2^ − 0.0440x + 1.1437	0.988	35	115
Scenic area & park	Scenic spot	y = 2.6741 ∙ 10^−7^x^3^ − 9.4410 ∙ 10^−5^x^2^ + 0.0040x + 0.9731	0.991	75	190
	Park	y = 3.0520 ∙ 10^−7^x^3^ + 0.0031x + 0.9760	0.995	65	175
Other	Large amenity	y = 2.2224 ∙ 10^−5^x^3^ − 0.0009x^2^ − 0.0309x + 1.1797	0.966	40	105
	Small amenity	y = 1.8519 ∙ 10^−5^x^3^ − 0.0011x^2^ − 0.0288x + 1.0653	0.984	30	105

The model used walking distance as the independent variables and used the percentage of users who reached the station within this distance as the dependent variable. The values of the dependent variable are between 0 and 1. When the walking distance is 240 m, almost no users are willing to use the station. So the feasible regions of the independent variables are between 0 and 240 m. When the percentage of users declined to 85%, that distance was chosen as the optimal distance. This result means that 85% of users are willing to choose the station if the distance between the station and building entrance/exit is the optimal distance [43]. The distance at which 15% of users are willing to choose the station was chosen as the tolerable distance [43]. This result means that only 15% of users are willing to choose the station if the distance between the station and building entrance/exit is the tolerable distance. The optimal distances and tolerable distances of 16 types of land uses were obtained based on the survey data and are listed in Table 5.

First, considering all land uses, the optimal distances ranged from 30 m to 75 m, and the tolerable distances ranged from 105 m to 190 m. For most land uses, the tolerable distances will be long if the optimal distance is long. Second, the optimal distances and tolerable distances differ based on the type of land use. The distance between the station and entrance/exit is longest for scenic areas and parks, shortest for other land uses, and a medium value for office areas and commercial districts. It likely because users who go to scenic areas and parks are not concerned with convenience and travel time. They are willing to walk longer distance. Third, the optimal distances and tolerable distances are proportional to the intensity of the land use. For different intensities of land uses of the same type, the optimal distances and tolerable distances are longer for a higher intensity of land use. It means the land uses with higher intensity hold greater attraction, users are willing to walk longer distances to these amenities. These results provide references for the planning, site selection and evaluation of public bicycle stations.

## Conclusions

Public bicycles are a sustainable and environmentally friendly mode of transportation that solves the “last kilometer” problem to help improve daily urban mobility. However, few studies on station location have been conducted from a micro perspective. Previous studies on station location located the stations only per zone without providing the exact location of the station in the zone. Long distances between the station and the building entrance/exit are a common problem for stations in Beijing and greatly affects a user’s willingness to use the stations. This paper studied the walking distance decay law of a user’s choice of station based on survey data, and a binary logistic model was established to analyze the factors influencing walking distance. Finally, the optimal distances and tolerable distances of different types and intensities of land uses were obtained. The conclusions can be applied to the planning and optimization of PBSs. A convenient system can strongly encourage individuals to use a public bicycle and can significantly increase daily bicycle use.

The walking distance decay law of a user’s station choice showed a clear pattern: a user’s willingness to use the station decreases with increasing walking distance. In addition, the distance decay laws of different types of land uses were different. For schools and hospitals, rapid decay began at a distance of 30 m, whereas rapid decay did not begin until 100 m for scenic areas and parks. Different distance decay laws were also observed for different land use intensities, and the decay speed was slower for a higher land use intensity.

Car ownership, travel purpose, use frequency, cycling time and satisfaction all significantly influence a user’s walking distance. Users who own a car accept a shorter walking distance, whereas users who use a public bicycle frequently and have a long cycling time accept a longer walking distance. Compared with users who are commuting and shopping, users who are traveling for leisure or sports accept a longer walking distance. Moreover, a shorter walking distance will improve user satisfaction.

The optimal distances and tolerable distances of 16 types and intensities of land uses were also provided. We suggest controlling the distance between the station and the building entrance/exit within the optimal distances. The distance cannot be longer than the tolerable distance, as few people will choose the station.

This study used actual walking distance to illustrate the users’ willingness to use PBS. It could be a preliminary study for future stated preferences study to elicit the willingness to use PBS against other alternative travel modes. As a future line of research to complement this study, we will design and conduct a stated preferences survey in order to obtain user preferences. We also will make a comparison between the walking alternative that was observed and other potential alternatives. Moreover, we will study the impact of intersections and mid-block crossing facilities on station site accessibility. A user’s station choice behavior under different crossing conditions should be analyzed, and a user’s station choice preference between nearby crossing facilities and nearby building entrances/exits should be explored.

## Supporting information

S1 Appendix Questionnaire. Questionnaire of the Use of Public Bicycle.
(DOCX)

S2 Appendix An example of data. The sheet contains the individual data points used in the binary logistic model.
(XLSX)
