# Supplementary material for: Determining the exact location of a public bicycle station—The optimal distance between the building entrance/exit and the station
Source: PLoS One. 2019 Feb 20;14(2):e0212478. doi: 10.1371/journal.pone.0212478 (PMC6382268; doi:10.1371/journal.pone.0212478)
Supplement: S1 Appendix — Questionnaire of the Use of Public Bicycle. (DOCX) [file pone.0212478.s001.docx]

公共自行车使用情况调查（in Chinese）

**1、您的性别：**A.男 B.女

**2、您的年龄：**A.24岁以下 B.25-34岁 C.35-44岁 D.45-54岁 E.54岁以上

**3、您的月收入：**A.1500元以下 B.1501-3000元 C.3001-5000元

D.5001-8000元 E.8001-15000元 F.15001元及以上

**4、您是否拥有私人小汽车：** A.有 B.无

**5、您本次出行的目的：**A.通勤/上下学 B.购物 C.休闲/锻炼 D.其他

**6、您使用公共自行车的频率为：**

A.基本每天 B.一周多次 C.一周一次 D.一个月一次 E.偶尔

**7、您本次使用公共自行车出行的模式：**

A.全程模式，起终点间全程使用公共自行车出行

B.换乘模式，起终点间使用公共自行车换乘公共交通

**8、您本次使用公共自行车的时间：**

A．小于10分钟 B.10-20分钟 C.20-30分钟 D.30-40分钟 E.40分钟以上

**9、您从租车点步行到建筑物的实际距离/从建筑物步行到租车点的实际距离：**

A.25米以内 B.26-50米 C.51-75米 D.76-100米 E.101-125米 F.126-150米

G.151-200米 H.201-250米 I.251-300米 J.300米以上

**10、您要从租车点前往____ 建筑物/您是从__ _建筑物出发到达租车点的。**

**11、您从租车点步行到建筑物的可接受最大距离/从建筑物步行到租车点的可接受最大距离：**

A.25米以内 B.26-50米 C.51-75米 D.76-100米 E.101-125米 F.126-150米

G.151-200米 H.201-250米 I.251-300米 J.300米以上

**12、对于公共自行车系统的总体满意度评价：**A.满意 B.一般 C.不满意

非常感谢您的支持！

Questionnaire of the Use of Public Bicycle

**1、Your gender：**A. male B. Female

**2、Your age：**A. below 24 B.25-34 C.35-44 D.45-54 E. above 54

**3、Your monthly income：**A. below 1500 yuan B.1501-3000yuan C.3001-5000yuan

D.5001-8000yuan E.8001-15000yuan F.above 15001yuan

**4、Your car ownership：** A own a car B.without a car

**5、What is your travel mode of this travel：**

A.work/study B.shopping C.leisure/sport D.others

**6、How often do you use public bicycle:**

A.everyday B.several times in a wee C.once a week

D.once a month E.less than once a month

**7、What is your travel mode of this travel:**

A. combine public bicycle with some other type of public transport

B.use public bicycle alone for journeys

**8、How long do you use public bicycle this travel：**

A．below 10 minutes B.10-20 minutes C.20-30 minutes

D.30-40 minutes E.above 40 minutes

**9、What is the actual waking distance between the building entrance/exit and the public bicycle station：**

A.below25 meters B.26-50 meters C.51-75 meters D.76-100 meters

E.101-125meters F.126-150 meters G.151-200 meters H.201-250 meters

I.251-300 meters J.above 300 meters

**10、You walked from public bicycle station to ____ building/You walked from _ _ building to public bicycle station.**

**11、What is the maximum waking distance between the building entrance/exit and the public bicycle station you can accept：**

A.below25 meters B.26-50 meters C.51-75 meters D.76-100 meters

E.101-125meters F.126-150 meters G.151-200 meters H.201-250 meters

I.251-300 meters J.above 300 meters

**12、What is your degree of satisfaction of public bicycle：**

A. satisfied B. generally C. dissatisfied

Thank you very much!
